# Synergistic Inactivation of Bacteria Using a Combination of Erythorbyl Laurate and UV Type-A Light Treatment

**DOI:** 10.3389/fmicb.2021.682900

**Published:** 2021-07-16

**Authors:** Yoonjee Chang, Jaewoo Bai, Hyunjong Yu, Pahn-Shick Chang, Nitin Nitin

**Affiliations:** ^1^Department of Food and Nutrition, Kookmin University, Seoul, South Korea; ^2^Department of Food Science and Technology, University of California, Davis, Davis, CA, United States; ^3^Division of Applied Food System, Major in Food Science & Technology, Seoul Women’s University, Seoul, South Korea; ^4^Department of Agricultural Biotechnology, Seoul National University, Seoul, South Korea; ^5^Center for Food and Bioconvergence, Seoul National University, Seoul, South Korea; ^6^Research Institute of Agriculture and Life Science, Seoul National University, Seoul, South Korea; ^7^Department of Biological and Agricultural Engineering, University of California, Davis, Davis, CA, United States

**Keywords:** erythorbyl laurate, UV type-A, synergism, RT-qPCR, antimicrobials, light activated antimicrobial agents

## Abstract

This study evaluated the synergistic antimicrobial activity of erythorbyl laurate (EL) and UV type-A (UVA). To investigate the mode of synergism, changes in gene expression and bacterial inactivation activity were examined. Individual treatments with EL (10 mM) or UVA caused a 1.9- or 0.5-log CFU/ml reduction respectively, whereas EL/UVA co-treatment resulted in a 5.5-log CFU/ml reduction in *Escherichia coli* viable cell numbers. Similarly, treatment with either EL (2 mM) or UVA for 30 min resulted in a 2.8- or 0.1-log CFU/ml reduction in *Listeria innocua*, respectively, whereas combined treatment with both EL and UVA resulted in a 5.4-log CFU/ml reduction. Measurements of gene expression levels showed that EL and UVA treatment synergistically altered the gene expression of genes related to bacterial membrane synthesis/stress response. However, addition of 10–50-fold excess concentration of exogenous antioxidant compared to EL reduced the synergistic effect of EL and UVA by approximately 1 log. In summary, the results illustrate that synergistic combination of EL and UVA enhanced membrane damage independent of the oxidative stress damage induced by UVA and thus illustrate a novel photo-activated synergistic antimicrobial approach for the inactivation of both the Gram-positive and Gram-negative bacteria. Overall, this study illustrates mechanistic evaluation of a novel photochemical approach for food and environmental applications.

## Introduction

Foodborne pathogens present a significant public health challenge, causing significant illness and deaths worldwide (Lynch et al., 2009; Giaouris et al., 2014; Callejon et al., 2015; Zhu et al., 2017; Bai et al., 2019). Particularly, foodborne pathogens such as *Listeria monocytogenes* and *Escherichia coli* O157:H7 are the major causes of foodborne diseases (Doyle, 1991; Zhu et al., 2017). Conventional approaches can reduce food safety risks but often rely on harsh chemicals such as sanitizers for sanitation of minimally processed food and food contact surfaces and/or extensive processing of food products such as thermal processing (Beuchat and Ryu, 1997; Sohaib et al., 2016). These conventional approaches may also significantly impact food quality and generate negative environmental effects (Gil et al., 2009). Additionally, extensive use of chemicals may also negatively influence workers’ health (Magauzi et al., 2011).

To improve sustainability of the food processing industry, including the processing of minimally processed products and the sanitation of food contact surfaces, it is necessary to develop novel and safe technologies to eliminate foodborne pathogens on food contact surfaces and food materials without significantly affecting both food quality and environment. The improvement in microbial inactivation has been demonstrated by simultaneous application of two or more antibacterial strategies at sub-lethal levels such as an antibacterial compound at a sub-lethal concentration and a mild physical treatment (Cossu et al., 2016; Bastarrachea et al., 2017; de Oliveira et al., 2017, 2018a). Based on this concept, the combination of UV type-A (UVA) light (wavelength from 320 to 405 nm) and a plant-derived bioactive has been investigated for the inactivation of foodborne pathogens (Kim et al., 2008; Zhao et al., 2013; Cossu et al., 2016). These studies indicated that a synergistic interaction between organic acids (gallic acid or lactic acid) or polyphenolic compounds and UVA light resulted in enhanced bacterial inactivation in diverse simulated food systems as well as biofilms (Cossu et al., 2016; de Oliveira et al., 2017). In addition, there are prior studies that have introduced the use of UVC light with other factors such as pH or temperature (Marquenie et al., 2003; Quintero-Ramos et al., 2004; Gayán et al., 2012, 2013). Synergetic treatment using ultrasound and natural compounds was also demonstrated with fresh produce. Synergistic antimicrobial efficacy was demonstrated by the use of ultrasound at 20 kHz or 1 MHz in combination with natural compounds (carvacrol, cinnamic acid, gallic acid, or lactic acid) to reduce bacterial counts of *Listeria innocua* and *E. coli* in wash water. Specifically, the combined treatment of ultrasound (1 MHz) and citral (10 mM) led to >1.5-log CFU/ml *E. coli* K12 reduction when compared with that of the individual treatments (Zhang et al., 2020). Most of these studies have focused on the phenomenological approach to characterize the synergistic antimicrobial activity and many studies have suggested the critical role of oxidative stress and cell membrane damage in inducing synergistic activity (Wang et al., 2019; Seok and Ha, 2021). These studies provide some insight into the mechanism of action but lack the evaluation of molecular changes, for instance, in the gene expression of target genes. Furthermore, changes in the expression of target genes will advance the fundamental understanding of the cellular and molecular impact of the synergistic combined treatments for bacteria. Additionally, many of these studies have focused on naturally existing phenolic compounds and represent only a small sub-set of food grade compositions. Therefore, expanding this synergistic antimicrobial concept to other food grade compositions could help select an optimal set of compounds.

In this study, erythorbyl laurate (EL, 6-O-lauroyl-erythorbic acid), an enzymatically esterified form of erythorbic acid (D-isoascorbic acid) and lauric acid, was selected as a model compound. Studies have demonstrated the potential of EL for diverse applications in food systems including as a multi-functional emulsifier with antioxidant and antimicrobial properties against the Gram-positive bacteria (Park et al., 2011, 2017). EL has antimicrobial activity against the Gram-positive bacteria such as *Staphylococcus aureus*, *Listeria monocytogenes*, and *Bacillus cereus*, whereas the Gram-negative bacteria are not significantly affected (Park et al., 2018). Synergistic combinations of EL with antibiotics (streptomycin, chloramphenicol, erythromycin, kanamycin, ampicillin, and nisin) have also been evaluated to improve the inactivation of *S. aureus* as EL may form membrane pores, thereby increasing the membrane permeability and enabling the antibacterial agents to easily disrupt the cytoplasmic membranes (Park et al., 2018). Thus, to improve the inactivation of both the Gram-positive and Gram-negative bacteria using EL, discover novel mechanisms for synergistic antimicrobial activity, accelerate the antimicrobial kinetics of EL, and reduce the effective concentration of EL required for bacterial inactivation, this study evaluated the synergistic combination of EL with a physical form of mild processing technology using a UVA light. To understand the underlying mechanisms of the observed synergistic antimicrobial activity, gene expression was evaluated to investigate the role of this synergistic interaction on oxidative stress, membrane stress response, and membrane synthesis genes. The target genes were selected based on a combination of published results (Buchmeier et al., 1997; Sperandeo et al., 2007; Kim et al., 2013b; Rao et al., 2013; Karas et al., 2015; Ezraty et al., 2017; Nakayama and Zhang-Akiyama, 2017; Yuan et al., 2018). The antimicrobial activity of EL has been attributed to an association with cell membranes (Park et al., 2018), and UVA has been linked to the generation of reactive oxygen species (ROS; Cossu et al., 2016; Yang et al., 2019). In summary, our results will expand the class of food grade compositions for the synergistic inactivation of bacteria using UVA technology and provide insights into the genetic factors influencing the synergistic antimicrobial activity of the selected compound with UVA light. Understanding mechanisms for achieving synergistic inactivation of bacteria will lead to development of novel class of synergistic antimicrobial combinations and potential applications in diverse conditions including food and environmental applications.

## Materials and Methods

### Bacterial Strains and Culture Conditions

*Escherichia coli* O157:H7 ATCC 700728 without Shiga toxin genes (*stx*1 and *stx*2) was generously provided by Dr. L. Harris, Department of Food Science and Technology, University of California, Davis. A plasmid containing the rifampicin-resistant gene was transformed into the original bacterium. A rifampicin-resistant *L. innocua* mutant [ATCC 33090; American Type Culture Collection (ATCC), Manassas, VA, United States] was provided by Dr. T. Suslow’s laboratory (University of California, Davis). Both bacterial species were grown in tryptic soy broth (TSB; Difco, Detroit, MI, United States) containing rifampicin (50 μg/ml) at 37°C with shaking at 150 rpm for 18–20 h. Enumeration was conducted on tryptic soy agar (TSA; Difco) media supplemented with rifampicin (50 μg/ml). *Escherichia coli* K-12 MG1655 (ATCC 700926) was used for the examination of gene expression as a strain with a complete genome sequence was needed; the strain was grown in TSB medium at 37°C with shaking at 150 rpm for 18–20 h.

### Preparation of Erythorbyl Laurate

Erythorbic acid (≥99.0%) and dodecanoic acid (lauric acid ≥ 99.0%) were purchased from Sigma-Aldrich Co. (St. Louis, MO, United States). Novozym 435 (i.e., lipase from *Candida antarctica* immobilized on a macroporous acrylic resin) was kindly provided by Novozymes (Bagsværd, Denmark) with a catalytic activity of 7,000 PLU/g (the activity of PLU refers to the millimoles of propyl laurate synthesized per min at 60°C). High-performance liquid chromatography grade acetonitrile, water, and acetic acid (J.T. Baker Co., Phillipsburg, NJ, United States) were filtered through a membrane filter (0.45 μm) before use. The enzymatic synthesis of EL was conducted in the gas–solid–liquid multiphase reaction system with slight modifications (Yu et al., 2019). After the addition of lauric acid (2.84 mol) into a reaction vessel, pre-incubation was conducted for 10 min to melt lauric acid at 60°C with sparging of N2 gas at 8.0 L/min. The reaction was initiated by adding erythorbic acid (1.42 mol) and immobilized lipase (120 mg/ml). The temperature was maintained at 60 ± 1°C during the entire reaction. After synthesis for 72 h, the reaction mixture was obtained by filtration with a porous glass filter (27.5 μm pore size) and purified by solvent separation (Park et al., 2017). Quantitative analyses were conducted on an LC-2002 system (Jasco, Tokyo, Japan) equipped with a C18 reverse-phase column (5 μm, 4.6 × 150 mm) and a UV detector (UV-2075; Jasco). EL was identified by its retention time, and the purity of EL was determined by the peak area at 265 nm according to the previous study (Park et al., 2011). The purity of EL used in this study was higher than 99.0%. A stock solution of EL at 500 mM concentration was prepared using 50% ethanol solution.

### Inactivation of *L. innocua* and *E. coli* O157:H7 After EL, UVA Light, and EL/UVA Treatment

Bacterial cells were grown to an early exponential phase, harvested (16,100 × g for 1 min), washed, and re-suspended in phosphate-buffered saline (PBS; pH 7.4; approximately 10^6^ CFU/ml). *Listeria innocua* was treated with EL (0–2 mM), UVA light (30 min), or a combination of both UVA and EL. *Escherichia coli* O157:H7 was treated with EL (0–10 mM), UVA (30 min), or a combination of both treatments. UVA exposure was conducted using a UVA chamber with four UVA lamps (320–400 nm, 18 W; Actinic BL, Philips, Holland) on the underside of the lid of a closed plastic box (Suncast Corporation, Batavia, IL, United States) as previously reported (Ercan et al., 2016; de Oliveira et al., 2018b). About 1 ml of each sample was placed in individual wells of a sterile 24-well flat-bottom polystyrene plate. The plate was placed at a distance of 8 cm from the UVA lamps at the center of the chamber. The average light intensity of the UVA was 40.8 ± 3.9 W m^−2^. After each treatment, the bacterial cells were harvested and enumerated. Additionally, bacterial inactivation assays were conducted as above to compare the antibacterial activities of EL, sodium erythorbate (SE), monolaurin (ML), and the mixture of SE and ML. Each compound (2 mM against *L. innocua* and 10 mM against *E. coli* O157:H7) was evaluated in combination with UVA treatment and control conditions (without UVA treatment) for the inactivation of selected bacteria.

To assess the involvement of oxidative stress in the synergistic inactivation of the combined treatments, EL was prepared at a final concentration of 2 mM against *L. innocua* and 10 mM against *E. coli* O157:H7 in either PBS or PBS supplemented with 100 mM thiourea, respectively. Supplementation with an antioxidant (thiourea) can quench ROS generated from UVA light treatment, preventing bacterial cells from being inactivated (Goswami et al., 2007; Sudzhaev et al., 2011). Bacterial cells were inoculated to 10^8^ CFU/ml in PBS or PBS supplemented with the antioxidant. The bacterial cells in UVA and EL/UVA co-treated groups were incubated under the UVA light for 30 min, and the bacterial cells in other groups were incubated without UVA exposure for 30 min. Viable bacterial cells were enumerated on TSA supplemented with rifampicin. The detection limit was 10 CFU/ml.

### Cell Permeability Assessment

Changes in cell permeability after exposure to EL in the presence or absence of UVA light were monitored as described previously (de Oliveira et al., 2017) with a slight modification, using SYTOX orange dye, which can penetrate only membrane-damaged cells. The fluorescence intensity of SYTOX orange increases upon binding to nucleic acids (Biggerstaff et al., 2006). First, bacteria (10^8^ CFU/ml) were treated with EL at room temperature or with UVA light for 30 min. Next, 1 ml of the suspensions was centrifuged at 16,000 × g for 2 min, and the pellets were resuspended in 1 ml of PBS. About 1 μl volume of a 5 mM stock solution of SYTOX orange was added (final concentration, 5 μM) and the sample was gently vortexed and incubated at room temperature for 15 min. The sample (150 μl) was transferred to a 96-well flat-bottom black polystyrene plate and the fluorescence signal intensity was assayed using a fluorescence plate reader (Tecan SpectraFluor Plus) with a 530 nm excitation filter and a 580 nm emission filter. The positive control was prepared by mechanically disrupting the cells using silica beads. Specifically, 1 ml of bacterial suspension diluted in PBS was vortexed with zirconia-silica beads at the highest speed setting for 10 min. Next, SYTOX orange was added to the lysed bacteria, and incubated for 15 min. The fluorescence intensity was considered indicative of damage to the bacterial cell membrane. The results were normalized to the value of the positive control and are expressed as percentages.

### RNA Isolation

To characterize changes in gene expression for *E. coli* K-12 MG1655 under EL, UVA light, and EL/UVA co-treatment conditions, reverse transcription-quantitative PCR (RT-qPCR) analyses were conducted. *Escherichia coli* K-12 MG1655 cultures were incubated until the early stationary phase and then treated with sub-lethal levels with EL (1 mM), UVA (15 min), or EL (1 mM)/UVA (15 min) conditions. Bacterial cultures were then stabilized with RNAprotect Bacteria Reagent (Qiagen, Hilden, Germany). RNA extraction was conducted using an RNeasy Mini Kit (Qiagen), and DNase digestion was conducted with a TURBO DNA-free™ Kit (Thermo Fisher Scientific, Waltham, MA, United States). The concentration and quality of extracted RNA were verified using a Nanodrop 1000 (Thermo Scientific Inc., Wilmington, DE, United States). Experiments were conducted with three biological replicates for each sample.

### RT-qPCR Assay

The expression of 17 genes (membrane synthesize genes, membrane stress response genes, and oxidative stress response genes) was examined by RT-qPCR. The gyrase A gene (*gyr*A) was used as an endogenous control for normalization within samples. Forward and reverse PCR primers for the target genes were newly designed based on the NCBI *E. coli* K-12 MG1655 strain complete genomic sequence (GenBank No. U00096.3) using Primer 3 software^1^ with the following criteria: amplicon size, 100–200 bp; calculated primer melting temperature, 53–62°C; GC content, 40–60%; and probabilities of primer-dimer/hairpin formation were minimized (Table 1). The template cDNA was synthesized from 1 μg extracted total RNA *via* reverse transcription using the Omniscript Reverse Transcription Kit (Qiagen) with random hexamers (Qiagen). The cDNA was used as a template for RT-qPCR with PowerUP™ SYBR® Green Master Mix (Thermo Fisher Scientific), with each RT-qPCR mix (total volume 20 μl) consisting of 2 μl cDNA, 2 μl each of forward and reverse primers (1 μM stock), 10 μl SYBR green master mix, and 4 μl nuclease-free water. The RT-qPCR reactions were conducted on an Open qPCR system (Chai Biotechnologies Inc., Santa Clara, CA, United States) with the following thermal cycling conditions: 95°C for 5 min (denaturation and polymerase activation) and 40 cycles of 95°C for 15 s and 55°C for 15 s (amplification). The specificity of the PCR was determined with melting curve analyses (72–95°C with a heating rate of 0.02°C/s). The relative changes in gene expression in EL, UVA, and EL/UVA co-treated cells compared with the untreated control were calculated using the 2^−ΔΔ^CT method (Rao et al., 2013).

**Table 1:** Primer sequences.

Gene	5'–3' sequence^a^	Function
*lptA*	F: CGGCGAACAAGGTAAAGAAG R: TTGCCAGTTCGTAGTGCATC	Participates in lipopolysaccharide (LPS) biogenesis
*lptB*	F: GAAATTGTCGGTCTGCTGGG R: CGACGGAAAATGGAGGCTTC	Forms membrane-related components for LPS transport
*pspD*	F: GGCCGGGCAAAAGGTAAAG R: GCCAGTTTATTAGCAGCCCG	Phage-shock protein related to membrane-altering stress
*ftsW*	F: TTGTTTGTGACTACGCTGGC R: TTGCGTTAACTGATAGCCGC	Translocates lipid II from the cytoplasm to the periplasmic machinery of peptidoglycan (PGN) assembly
*uppP*	F: ATTGGGTGTGGTCGAAGGAT R: TTCAAAGGTTTTCGCCGTGT	Related to the biosynthesis of cell wall components including PGN and LPS
*mraY*	F: ACCATGGGCGGGATTATGAT R: AACACCGTAACCTACCAGCA	Catalyzes the first membrane step of PGN synthesis
*murG*	F: ATCTCTGGTCTGCGTGGAAA R: TGACACGTAGCCTCCCATAC	Involved in PGN synthesis, transfers an *N*-acetylglucosamine moiety to lipid I for lipid II production
*uppS*	F: AATACCGGTCTGACGCTGAA R: GGGCCAGTTCATGCATACAG	Undecaprenyl pyrophosphate synthase gene
*soxR*	F: GTATCGGCGCTGCATTTCTA R: CGGAATGCCAATACGCTGAG	Transcriptional activator of oxidative stress regulon
*soxS*	F: ATCAGACGCTTGGCGATTAC R: ACATAACCCAGGTCCATTGC	Regulation of superoxide response regulon
*grxA*	F: ACTGTGTGCGTGCAAAAGAT R: CCCATGCAGCAAAATCGGTA	Oxidative damage repair enzyme
*msrB*	F: ACTGTTTGATCTGCGATGCC R: GGAAGACATGCCCCAGATGG	Oxidative damage repair enzyme
*pqiA*	F: GTTCCCGCGTTTTGTCTGAT R: AACGAAACTGACCAGCACAC	Paraquat-inducible gene induced by oxidative agents
*pqiB*	F: GATTCGTATCGAGCCAGAGC R: CGGTTATTGCAGGCGTATTT	Paraquat-inducible gene induced by oxidative agents
*slyA*	F: AAACTGACGGAAAAGGCAGA R: CCCTTTGGCCTGTAACTCAA	Required for resistance to oxidative stress
*adeD*	F: TAACGGTGCCAGTTTTACCC R: AGTTTATCGAGCAGCGCATT	Induced upon oxidative stress
*dps*	F: GCGCTAACTTCATTGCCGTA R: CCTGAACGTTGTGGATGTCC	Protects DNA from oxidative damage
*gyrA*	F: CTGCTGGTGAACGGTTCTTC R: ACGACCGTTAATGATTGCCG	Housekeeping gene

a F, forward.

R, reverse. All primers were designed for this study.

### Statistical Analyses

All of the experiments were conducted in triplicate. The bacterial population and gene expression levels were statistically analyzed by SAS 9.4 (SAS Institute Inc., Cary, NC, United States). ANOVA followed by Tukey’s multiple comparison test (95% CI) was conducted. The data are presented as means and SDs. A value of *p* < 0.05 was considered statistically significant.

## Results and Discussion

### Synergistic Inactivation of *L. innocua* and *E. coli* O157:H7 Using a Combination of EL and UVA Light

**Figure 1:**
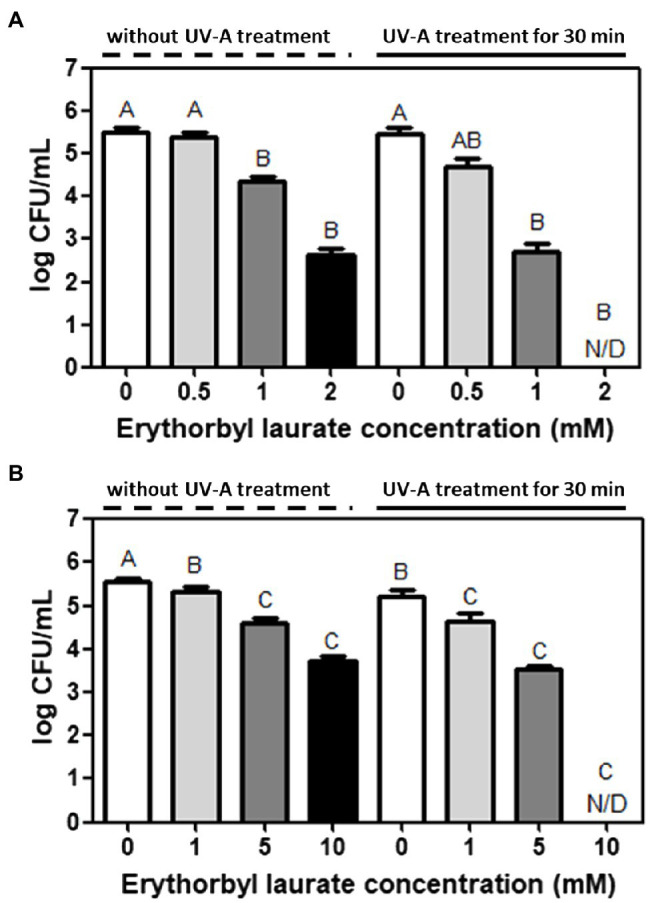
Synergistic bacterial inactivation by erythorbyl laurate (EL) and UV Type-A (UVA) light against **(A)** *Listeria innocua* and **(B)** *Escherichia coli* O157:H7. The mean values from three independent measurements are shown. N/D, not detected. Different uppercase letters indicate significant differences (*p* ≤ 0.05).

The bacterial counts of *L. innocua* and *E. coli* O157:H7 were synergistically reduced using a combined treatment with EL and UVA light compared with that of individual treatments of bacterial cells with either EL or UVA light (Figure 1). An initial screening test was conducted to determine the effective inhibitory concentration of EL (2 mM) against *L. innocua*. The combination of EL (2 mM) and UVA was also tested against *L. innocua* using the same experimental method as above. Specifically, the combined treatment of EL (2 mM) and UVA light reduced *L. innocua* cells by more than 5.4-log CFU/ml within 30 min compared to the individual-treatment groups (2.8-log CFU/ml reduction with the *L. innocua* 2 mM EL-treated group and 0.1-log CFU/ml reduction in the UVA light-treated group; Figure 1A). Similarly, an initial screening test was conducted to determine the effective inhibitory concentration of EL (10 mM) against *E. coli*. The combination of EL (10 mM) and UVA was also tested against *E. coli* O157:H7 using the same experimental method as above (Figure 1B). More than 5.4-log CFU/ml reduction in counts was achieved within 30 min using the combination of EL (10 mM) and UVA light compared with the 1.9-log CFU/ml reduction in the 10 mM EL-treated group and 0.5-log CFU/ml reduction in the UVA-treated group. EL was previously revealed to only have antibacterial efficacy against the Gram-positive bacteria (Park et al., 2018). However, this study showed that the combination of EL and mild physical treatment (UVA light) could rapidly reduce both the Gram-positive and Gram-negative bacteria. Previously, microbial inactivation with EL had only been confirmed based on minimum inhibitory concentration (MIC) and minimum bactericidal concentration against various bacterial strains at 37°C for 12 h (MIC, 0.88 mM against *S. aureus* ATCC 12692; 0.73 mM against *B. cereus* ATCC 13061; 0.58 mM against *L. monocytogenes*; Park et al., 2018). In this prior study, the synergistic antibacterial test with antibiotics and EL was evaluated by the checkerboard test at 37°C for 12 h, and consequently, the fractional inhibitory concentration (FIC) index was calculated (∑FIC: 1.25 with potassium sorbate, 1.25 with cloxacillin, and 0.75 with rifampicin; Park et al., 2018). Compared with the results of the prior study, EL treatment in combination with UVA light achieved significant inactivation of both the Gram-positive and the Gram-negative bacteria in a short treatment time. This enhanced inactivation may have applications in the food processing industry, including sanitation and pasteurization of food and food contact surface, as well as applications in biomedical and environmental microbial controls.

### Comparison of Bacterial Inactivation Activities Among EL, SE, ML, and SE/ML

Erythorbyl laurate is enzymatically synthesized by a combination of lauric acid and erythorbic acid. To understand the contributions of individual compounds for the synergistic antimicrobial activity, we selected sodium salt of erythorbic acid (SE) and ML. Monolaurin, a monoglyceride of lauric acid, is a lipophilic moiety of EL and has antibacterial activity against various bacteria (Lieberman et al., 2006; Altieri et al., 2009; Park et al., 2018). SE is a sodium salt of erythorbic acid and is mainly used as an antioxidant in food products (Hsu and Sun, 2006). In this test, we used the same concentration levels of individual compounds as the concentration level of EL used in this study. In addition, we also evaluated the physical mixture of monolaurin and SE and compared the results with EL both with and without UVA treatment.

**Figure 2:**
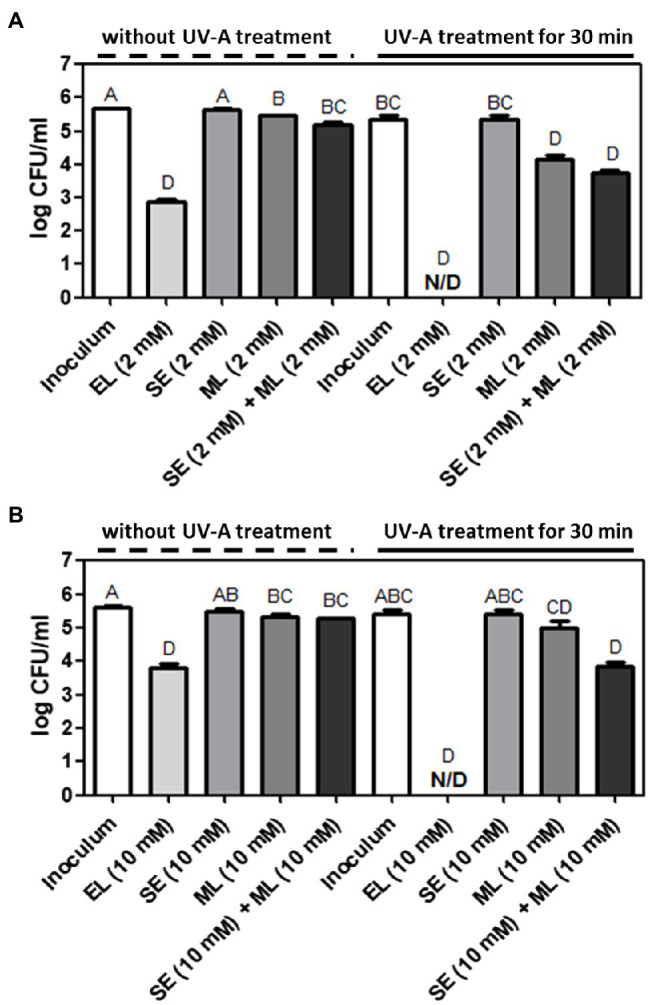
Comparison of antibacterial activities among EL, sodium erythorbate (SE), monolaurin (ML), and SE/ML mixture with or without UVA light treatment against **(A)** *L. innocua* and **(B)** *E. coli* O157:H7. The mean values from three independent measurements are shown. N/D, not detected. Different uppercase letters indicate significant differences (*p* ≤ 0.05).

Erythorbyl laurate (2 mM) induced a 5.5-log CFU/ml reduction of *L. innocua* cells in combination with UVA treatment for 30 min. This was a significantly (*p* ≤ 0.05) higher level of bacterial inactivation than that observed in the groups treated with 2 mM SE (0.0-log CFU/ml reduction), 2 mM ML (1.6-log CFU/ml reduction), or a combination of 2 mM SE/2 mM ML (1.7-log CFU/ml reduction) in the presence of UVA light (30 min) treatment (Figure 2A). Similarly, the EL (10 mM) + UVA-treated group reduced in *E. coli* O157:H7 population by 5.6-log CFU/ml, which was also a significantly higher level (*p* ≤ 0.05) of antibacterial activity than that observed in the groups treated with 10 mM SE (0.1-log CFU/ml reduction), 10 mM ML (0.4-log CFU/ml reduction), or the combination of 10 mM SE/10 mM ML (1.6-log CFU/ml reduction) in the presence of UVA light (30 min; Figure 2B). Thus, the enzymatically combined form of erythorbic acid and lauric acid had stronger antibacterial activity than the individual compounds (SE or ML) or their physically combined mixture in the presence of UVA light. The enhanced membrane partitioning activity of the EL compound in the cell membrane combined with UVA stress may result in a synergistic inactivation of target bacteria and the observed increase in antimicrobial activity. The UVA and EL combination may also generate local oxidative damage in the membrane or induce oxidative stress in the cell cytoplasm as suggested by the use of an amphiphilic antimicrobial peptide in combination with UVA light (Yang et al., 2019).

### Evaluation of Antibacterial Effects of EL, UVA Light, and EL/UVA Co-treatment Against *L. innocua* and *E. coli* O157:H7 in the Presence of Antioxidants

**Figure 3:**
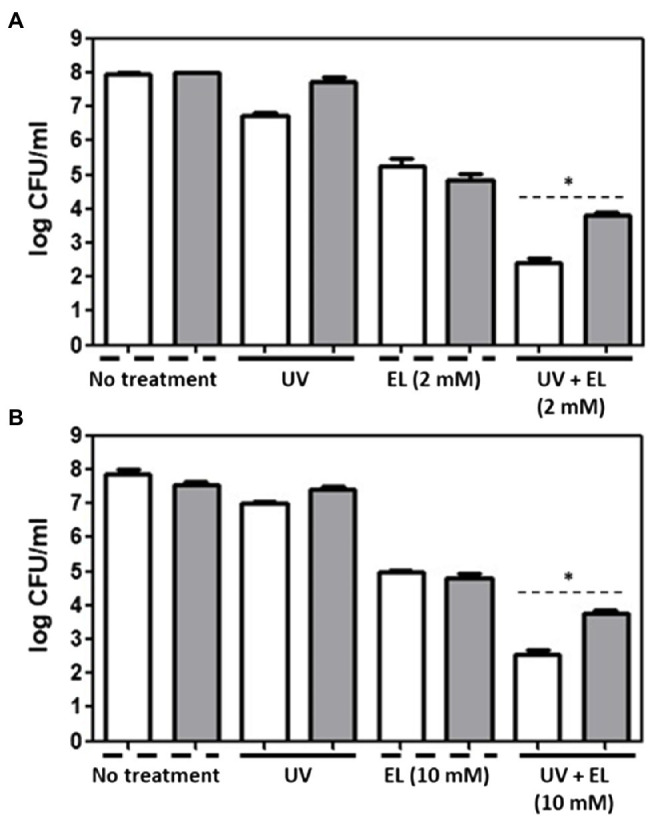
Evaluation of antimicrobial effects of UVA and EL. UVA light-, EL-, and UVA/EL-treated **(A)** *L. innocua* and **(B)** *E. coli* O157:H7 cells in the presence of 100 mM thiourea (gray bar), respectively, compared with the negative control without antioxidant (white bar). The mean values from three independent measurements are shown. Asterisk (*) indicates significant difference between thiourea-untreted and -treated groups (*p* ≤ 0.05).

One pathway through which synergistic interactions between EL and UVA may occur is the enhanced generation of ROS. To evaluate the role of ROS in the synergistic interactions, we added exogenous antioxidants to attenuate ROS-related antimicrobial activity. Incubation with exogenous antioxidant (thiourea, 100 mM) led to a slight but statistically significant reduction in the antibacterial synergy between EL and UVA (Figure 3); in the presence of an exogenous antioxidant, >4-log CFU/ml of bacteria were inactivated compared with bacterial numbers in the controls and in the absence of an exogenous antioxidant, >5-log CFU/ml of bacteria were inactivated compared with bacterial numbers in the controls due to different initial cell concentration used (Figure 1). We selected 8 log of bacteria as our initial inoculum as our goal in the antioxidant quenching assay was to have a survival population of bacteria after the synergistic treatment as a lack of detectable cells with synergistic treatments makes it difficult to quantify the exact inhibitory effect of the antioxidants on the synergistic treatment. Notably, the level of the exogenous antioxidant was at least 10-fold higher than the level of the EL used in this study and had only a limited suppressive effect on the synergistic antimicrobial activity. In contrast, results from our recent study demonstrate that addition of thiourea can completely quench the synergistic interaction of phenolic acids (gallic acid and dihydroxy benzoic acid) with UVA (4–5 log inhibition; de Oliveira et al., 2021). Thus, based on this comparison, it is clear that the exogenous antioxidant only has a marginal impact on the synergistic interaction of EL and UVA. We also tested glutathione as an exogenous antioxidant, but there was no significant reduction in the synergistic antimicrobial activity of the combination of EL and UVA (data not shown). Overall, these results suggest the limited role of ROS generation in the synergistic antimicrobial activity. This observation is in contrast to various previous studies that have highlighted the role of ROS in the synergistic activity of various polyphenolic and other diverse plant derived bioactives (Cossu et al., 2016; de Oliveira et al., 2017, 2018a,b). To understand the mode of synergistic interactions between EL and UVA, we then evaluated the changes in the expression of target membrane, metabolic activity, and oxidative stress response genes.

### Effects of EL and UVA Light on Bacterial Cell Membrane Permeability

**Figure 4:**
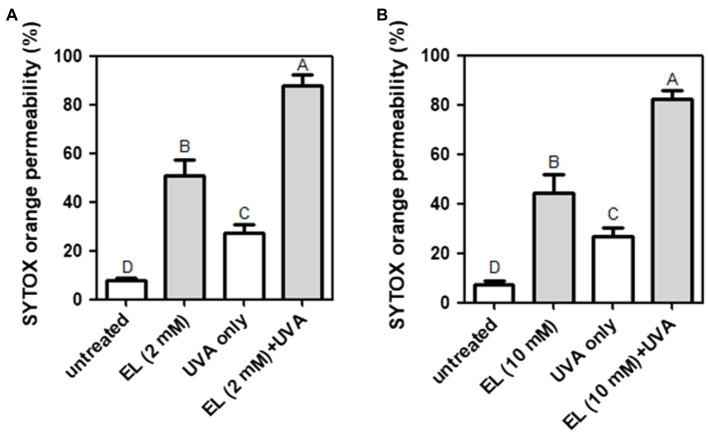
Permeation of SYTOX orange into **(A)** *L. innocua* and **(B)** *E. coli* O157:H7 cells treated with EL with or without UVA light (30 min). Means of three independent measurements are shown. Different uppercase letters indicate significant differences (*p* ≤ 0.05).

The effects of EL and UVA light on bacterial cell membrane permeability were examined. The untreated negative control showed little permeation of SYTOX orange (7.9%). Treatment with UVA light (27.3%) and 2 mM EL (51.5%) significantly (*p* ≤ 0.05) increased the permeability of *L. innocua* cells (Figure 4A). The combination of UVA light with 2 mM EL (87.9%) significantly (*p* ≤ 0.05) increased cell membrane permeability compared to single treatments (Figure 4A). The combination of 2 mM EL and UVA light exerted a synergistic effect on cell membrane permeability.

Treatment with EL (10 mM) without UVA light increased the cell membrane permeability of *E. coli* O157:H7 by 44.4% (Figure 4B), whereas treatment with EL (10 mM) with UVA light resulted in a significant (*p* ≤ 0.05) and synergistic increase in cell membrane permeability (82.1%). Therefore, permeabilization of the bacterial cell membrane may be responsible for the synergistic antibacterial effect of EL and mild heating against *L. innocua* and *E. coli* O157:H7.

### Gene Expression Changes in Response to EL, UVA Light, and EL/UVA Co-treatment

**Figure 5:**
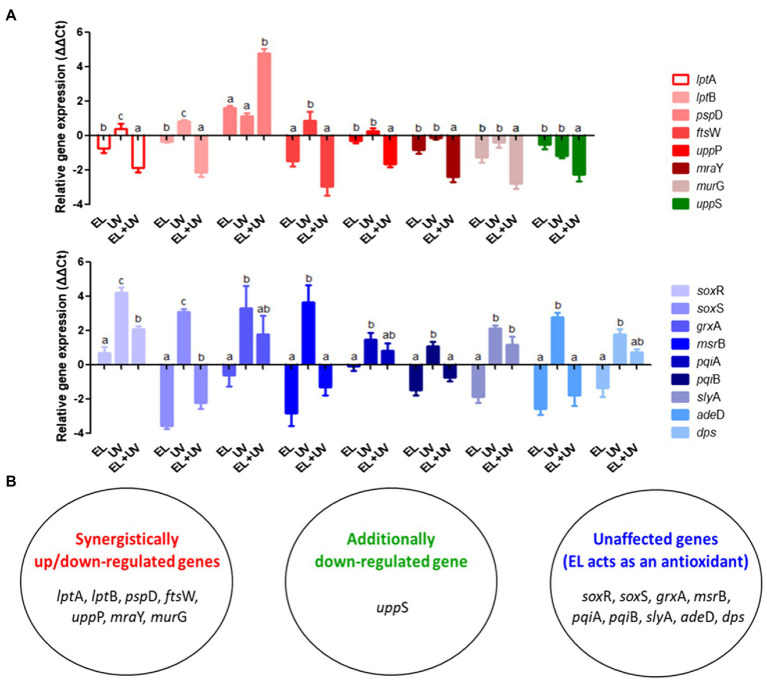
**(A)** Expression levels of genes related to membrane synthesis/stress and oxidative stress responses in *E. coli* MG1655 cells determined by reverse transcription-quantitative PCR (RT-qPCR). Bacterial cells were non-adapted (control) or adapted to sublethal EL concentrations (1 mM), UVA light (UV, 15 min), or EL/UVA co-treatment (EL + UV, 1 mM EL, and 15 min UV treatment). **(B)** Grouping of the synergistically affected, additively affected, or unaffected genes. The mean values from three independent measurements are shown. Different lowercase letters indicate significant differences (*p* ≤ 0.05).

Supplementary Figure S1 validates synergistic inactivation of *E. coli* MG1655 cells using a combination of EL and UVA. The treatment time was 15 min to avoid complete inactivation of bacteria. To examine how the expression of stress-related genes responded to EL, UVA, and EL/UVA induced-stress, RT-qPCR analyses were conducted. RT-qPCR approach was selected as the genes involved in membrane stress, cell envelope synthesis, and oxidative stress have been well characterized in prior studies (González-Guerrero et al., 2007; Pati et al., 2014). The set of genes that were significantly upregulated or downregulated when *E. coli* MG1655 was treated with a sub-lethal concentration of EL, UVA, or EL/UVA are shown in Figure 5 and were classified according to their expression patterns in the cell. Genes involved in membrane stress (*psp*D) or cell envelope synthesis (*lpt*A, *lpt*B, *fts*W, *upp*P, *mra*Y, and *mur*G) were synergistically upregulated or downregulated in the EL/UVA co-treated group compared with expression in either the EL- or the UVA-treated group (Figure 5; Sperandeo et al., 2007; Kim et al., 2013b; Ruiz, 2015; Liu and Breukink, 2016; Yuan et al., 2018). Synergistic combination induced upregulation or downregulation of expression was defined as more than a 4-fold change in the level of gene expression than that induced by cumulative changes of the individual treatments. One peptidoglycan (PGN) biosynthesis gene (*upp*S) was also downregulated in the EL/UVA co-treated group compared with the expression in either the EL- or UVA-treated group (Figure 5; Liu and Breukink, 2016). In this case, the downregulation effect was additive in nature as the level of the change in expression of this gene was less than 4-fold than that of the cumulative change induced by the individual treatment. The oxidative stress-related genes (*sox*R, *sox*S, *grx*A, *msr*B, *pqi*A, *pqi*B, *sly*A, *ade*D, and *dps*) were the most upregulated genes in the UVA-treated group (Figure 5; Buchmeier et al., 1997; Ye et al., 2013; Nakayama and Zhang-Akiyama, 2017; Yuan et al., 2018). UVA light generates ROS and damages both bacterial DNA and cellular components (Kumar et al., 2004; Bosshard et al., 2010). UVA can also oxidize intracellular proteins and cell membranes (Kim et al., 2013a; Santos et al., 2013). Although oxidative stress-related genes were more highly expressed in the UVA-treated condition, the expression of these genes was significantly downregulated in the EL/UVA-treated group. This could be attributed to EL acting as an antioxidant and suppressing oxidative stress in the cell (Park et al., 2017). Therefore, based on the gene expression analysis, EL/UVA co-treatment led to synergistic bacterial inactivation mainly because of the enhanced severity of bacterial membrane stress and damage caused by both treatments. Additionally, ROS generated by UVA may have exacerbated the damage to bacterial cells, although this effect would be reduced by the antioxidant properties of EL, suggesting that ROS have a minimal role in the synergistic reduction of bacterial cells.

Although several studies have reported synergistic activity of food grade compounds such as lauric arginate or fumaric acid with UVA light (Yang et al., 2019; Jeon and Ha, 2020), to the best of our knowledge, this is the first study to evaluate changes in bacterial gene expression induced by the synergistic antimicrobial interaction of the model compound with the selected mild physical process. Characterizing the mechanism of synergistic interactions using biochemical evidence, such as antioxidant quenching assays, changes in membrane permeability, and metabolic activity of cells, is often challenging (Yang et al., 2019). Conventionally, synergistic interactions among food grade compounds and UVA light have been attributed to classical photodynamic pathways involving the generation of diverse ROS (type I and type II pathways) as the predominant factor for bacterial inactivation (Garcia-Diaz et al., 2016). This study incorporated the analysis of changes in gene expression to suggest a mechanism to achieve synergistic inactivation of bacteria using a combination of chemical compounds and UVA light. Unlike prior studies, this study focused on enhancing the membrane damage induced by the synergistic combination of EL and UVA light. Since the synergistic activity was achieved without significant ROS generation, this provides an advantage for application of this technology for food systems as ROS generation can lead to potentially undesirable oxidation in some food products. Previous studies have indicated that UVA light has the potential to induce bacterial cell membrane damage, although the extent of damage was often to limited to induce a significant reduction (>2-log CFU/ml) in bacterial cell viability (Ha et al., 2011). Similarly, the treatment of bacterial cells with EL was also shown to induce cell membrane damage, but there was a limited reduction in cell viability, and this was only effective against the Gram-positive bacteria (Park et al., 2018). The results of our study demonstrate that combining EL and UVA treatment synergistically and significantly induces membrane damage and alters the gene expression profiles of membrane synthesis and repair genes.

## Conclusion

In this study, a novel synergistic antimicrobial approach was investigated, and an underlying genetic mechanism was evaluated. EL and UVA co-treatment resulted in synergistic bacterial inactivation in both the Gram-positive and Gram-negative bacteria. Mechanisms for the antimicrobial effects of EL and UVA were proposed based on changes in the expression levels of various stress response genes. Membrane synthesis/stress genes were synergistically regulated in the EL/UVA co-treated group. Oxidative stress genes were upregulated under the UVA treatment condition; however, their expression decreased in the EL/UVA co-treated group as EL may act as an antioxidant. We conclude that EL did not cause oxidative damage but mainly induced damage to the bacterial cell membrane. Additionally, UVA not only caused oxidative damage to cellular components but also damaged the cell membrane. The combination of a safe antimicrobial agent and a common physical treatment may have practical applications in sanitizing procedures in the food and biomedical industries. Furthermore, this study provides insights into changes to gene expression that will aid the development of novel treatments that combine both chemical and physical components. Future studies may be designed to expand the genetic and metabolomic analysis to further characterize the response of bacterial cells to synergistic treatments. In addition, future studies may evaluate the translation of synergistic processing of food products using a combination of EL and UVA as well as discovery of related food grade compounds with synergistic antimicrobial activity. These translational studies will complement the mechanistic approach developed in this study and all together will advance synergistic processing in food industries.

## Data Availability Statement

The raw data supporting the conclusions of this article will be made available by the authors, without undue reservation.

## Author Contributions

YC: formal analysis, investigation, and writing original draft. JB: conceptualization, formal analysis, and writing and editing original draft. HY: sample preparation and performing experiment. P-SC: data analysis. NN: conceptualization, resources, writing original draft, review, and editing, and supervision. All authors contributed to the article and approved the submitted version.

### Conflict of Interest

The authors declare that the research was conducted in the absence of any commercial or financial relationships that could be construed as a potential conflict of interest.

## Footnotes

^1^https://bioinfo.ut.ee/primer3-0.4.0/
