# Supplementary material for: Synergistic Inactivation of Bacteria Using a Combination of Erythorbyl Laurate and UV Type-A Light Treatment
Source: Front Microbiol. 2021 Jul 16;12:682900. doi: 10.3389/fmicb.2021.682900 (PMC8322444; doi:10.3389/fmicb.2021.682900)
Supplement: Supplementary file 1 [file Table_1.DOCX]

Supplementary Material


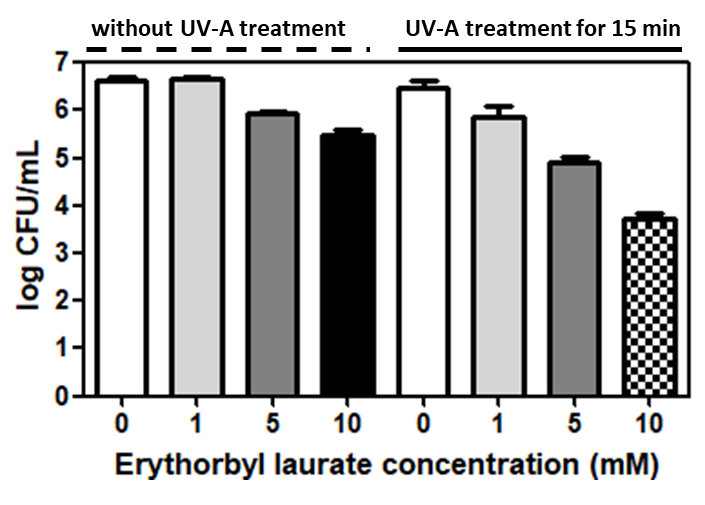


**Supplementary Figure 1**. Synergistic bacterial inactivation by erythorbyl laurate (EL) and UVA light against *E*. *coli* MG1655 after 15 min of treatment. The mean values from three independent measurements are shown. The results show that with 15 min of treatment time, the synergistic combination of UVA and EL enhances the level of bacterial inactivation compared to EL treatment alone and this treatment time of 15 min was selected to avoid complete inactivation of bacteria.
